# Honey bee queen health is unaffected by contact exposure to pesticides commonly found in beeswax

**DOI:** 10.1038/s41598-021-94554-1

**Published:** 2021-07-26

**Authors:** Alison McAfee, Joseph P Milone, Bradley Metz, Erin McDermott, Leonard J Foster, David R Tarpy

**Affiliations:** 1 Department of Applied Ecology, North Carolina State University, Raleigh, NC 27695 USA; 2 Department of Biochemistry and Molecular Biology, Michael Smith Laboratories, University of British Columbia, Vancouver, BC V6T1Z4 Canada

**Keywords:** Proteomics, Metabolism, Environmental impact, Entomology

## Abstract

Honey bee queen health is crucial for colony health and productivity, and pesticides have been previously associated with queen loss and premature supersedure. Prior research has investigated the effects of indirect pesticide exposure on queens via workers, as well as direct effects on queens during development. However, as adults, queens are in constant contact with wax as they walk on comb and lay eggs; therefore, direct pesticide contact with adult queens is a relevant but seldom investigated exposure route. Here, we conducted laboratory and field experiments to investigate the impacts of topical pesticide exposure on adult queens. We tested six pesticides commonly found in wax: coumaphos, tau-fluvalinate, atrazine, 2,4-DMPF, chlorpyriphos, chlorothalonil, and a cocktail of all six, each administered at 1, 4, 8, 16, and 32 times the concentrations typically found in wax. We found no effect of any treatment on queen mass, sperm viability, or fat body protein expression. In a field trial testing queen topical exposure of a pesticide cocktail, we found no impact on egg-laying pattern, queen mass, emergence mass of daughter workers, and no proteins in the spermathecal fluid were differentially expressed. These experiments consistently show that pesticides commonly found in wax have no direct impact on queen performance, reproduction, or quality metrics at the doses tested. We suggest that previously reported associations between high levels of pesticide residues in wax and queen failure are most likely driven by indirect effects of worker exposure (either through wax or other hive products) on queen care or queen perception.

Queens are normally the sole reproductive female within a honey bee colony and the reproductive status of a queen directly influences the colony’s overall health; however, queen quality can be compromised by environmental stressors^1,2^. A queen can lay up to two thousand eggs/day in the spring^3^, and a queen’s egg-laying rate directly influences the number of worker offspring produced in a colony. Furthermore, queens invest substantial metabolic resources into maintaining viable spermatozoa within their spermathecae^4–6^. Honey bee queens take part in nuptial flights soon after their emergence as adults and store the spermatozoa (sperm) they acquire for the duration of their lives^3^. Once exhausted of viable sperm, queens can no longer produce female worker offspring and are replaced by the colony^4^. Colony losses are consistently attributed to poor queens by beekeepers^7^, and pesticide exposure is an environmental factor that is linked to declines in reproductive health and longevity^1,8–14^.

While fulfilling agricultural pollination services, honey bees are often exposed to agrichemicals, including pesticides^10,15–17^. Moreover, the transport of commercial colonies to multiple farms within a single pollination season can further increase the potential for contact with pesticides^10^, as pesticide exposure risk varies among different land uses and crops^17,18^. Foragers collect and store residue-containing food (nectar and pollen) inside the hive; therefore, pesticides present in the ambient landscape are commonly found as chemical residues in beeswax and food resources^10,17,19^. Additionally, miticides applied directly into the hive as a control measure against the parasitic *Varroa* mite (*V. destructor*) are found as chemical residues in wax^10,17^. While both beekeeper-applied miticides and agrichemicals are detected in hive matrices from commercial colonies, miticides tend to be the most dominant residues^10^. In both cases, however, the effects of in-hive exposures to these residues—individually and in combination—on honey bee reproductive health is an understudied aspect of honey bee toxicology^20^. Previous research has shown that the presence of a realistic pesticide cocktail in the wax of queen rearing cups had no discernable effect on queen biology; however, bees in colonies fed pollen containing field-realistic levels of pesticides present in bee bread produce less royal jelly and are less able to rear queens compared to untreated colonies^11,21^. Queens reared in exposed colonies subsequently performed more poorly than control queens^11^, demonstrating that even indirect exposure during queen development can be harmful. Direct exposure of queens to neonicotinoids during development also has adverse effects^22^; however, neonicotinoids are not commonly found within the wax matrix of commercial colonies^10,17^.

While residues in food resources can result in oral exposure or altered jelly secretions, chemicals present in wax could also be delivered through contact with the cuticle. Beeswax is the primary nest substrate on which bees live and store food, and beeswax is largely comprised of complex esters and fatty acids, produced by the workers glandular secretions^23^. The composition of beeswax is conducive for accumulating lipophilic molecules, including some pesticides^24,25^. As a result, beeswax from commercial honey bee colonies may contain a large number of chemical residues, some at high concentrations. A large-scale, multi-pesticide residue screening survey (n = 108) found an average of 10 different pesticide residues in a given sample, with miticides (coumaphos, tau-fluvalinate, and 2,4-DMPF—an amitraz degradation product) and insecticides (fipronil, deltamethrin, fenpropathrin, and permethrin) among the most toxic^10^.

The likelihood for a chemical to result in harm (risk) depends on both its exposure and toxicity. The Hazard Quotient (HQ) is a risk estimation approach for multi-pesticide mixtures, calculated by dividing the exposure (amount) of each pesticide by its respective toxicity (LD_50_), then summing the HQs for each pesticide to estimate the cumulative hazard^26^. While it has its limitations^27^ (e.g. the approach does not account for pesticide interactions, as in most cases these are not well-defined, nor pesticide transference efficiency of topical exposure), it is nonetheless a useful framework to approximate hazard for pesticide blends^10,14,15,17,19,28^. It has been previously reported that colonies containing wax with higher HQs had a higher incidence of queen events, with queenright colonies having an average HQ around 1500 and colonies exhibiting the loss of a queen having an HQ of nearly 3500^10^. High exposures to these miticides in beeswax during development have been shown to influence queen health, including effects on pupal weight, sperm count, and sperm viability^8,29^. Furthermore, topical treatment of queens with imidacloprid, a neonicotinoid insecticide, has been previously shown to reduce stored sperm viability^1^; however, this is not one of the most common pesticide residues found within hives^10,17^ especially in wax because the compound is hydrophilic.

Aside from mating flights early in life, queens typically remain within a colony, but they may still be exposed to in-hive pesticide residues from food (through the workers) and wax (the nest substrate). Indeed, some evidence suggests that topical exposure could affect queen sperm health and brood offspring survival^1,30^. Queen fertility could also theoretically be impacted indirectly via mating with compromised drones, which can have reduced fertility or sperm quality after exposure to pesticides, as has been previously reported for some neonicotinoids^31,32^. Here, we were interested in examining direct queen-pesticide interactions via topical exposures, which is a surprising gap in the literature. While topical exposure of workers has been traditionally employed when screening for honey bee pesticide toxicity (See Tier I assessment, USEPA 2014^33^), with few exceptions^1,30,34,35^, the effects of contact exposures on individual queens are seldom investigated. Queens could experience topical absorption through contact with residue-containing wax while walking and laying eggs, although it is important to note that the in-hive dynamics of pesticide transference and availability from beeswax has not yet been defined, and lifetime exposures for honey bee queens is therefore not known. Here, we topically exposed queens to pesticides, both individually and as a cocktail, using previously reported in-hive, relative concentrations documented in beeswax, applied at a range of concentrations. This work examines the risks these residues may pose to adult queen reproductive health in both laboratory and field conditions.

## Results

In order to test if topical exposures of different pesticides commonly found in wax affected queen quality metrics, we exposed queens to varying doses of six pesticides (coumaphos, fluvalinate, 2,4-DMPF, chlorothalonil, chloropyrifos, and atrazine) as well as a complete cocktail, then measured queen mass, sperm viability, as well as other morphometrics associated with reproductive quality (head width, thorax width, spermatheca width, and sperm counts). The doses ranged from 1× to 32×, where x is the median wax concentration for that compound as reported by Traynor et al.^10^ (Table 1). Control queens were exposed to an equal volume of solvent control (acetone).

**Table 1:** Applications of each pesticide in terms of hazard quotients (HQ) and parts per billion (ppb).

Pesticide	LD_50_	T1 (1 ×)		T2 (4 ×)		T3 (8 ×)		T4 (16 ×)		T5 (32 ×)	
		ppb	HQ	ppb	HQ	ppb	HQ	ppb	HQ	ppb	HQ
Fluvalinate	4.32	4310	998	17,240	3991	34,480	7981	68,960	15,963	137,920	31,926
Coumaphos	5.93	943	159	3772	636	7544	1272	15,088	2544	30,176	5089
2,4-DMPF	75	304	4.1	1216	16.2	2432	32.4	4864	64.9	9728	129.7
Chlorothalonil	111	361	3.3	1444	13.0	2888	26.0	5776	52.0	11,552	104.1
Chlorpyrifos	0.08	2.7	35.4	10.8	141.7	21.6	283.5	43.2	566.9	86.4	1,133.9
Atrazine	98.5	5.4	0.05	21.6	0.22	43.2	0.44	86.4	0.88	172.8	1.75
Cocktail	–	–	1200	–	4798	–	9596	–	19,192	–	38,384

Each treatment is expressed as a multiplication of the corresponding amounts quantified from commercially surveyed wax, where x = the median^10^. LD_50_ values are for workers and were sourced from Traynor et al. 2016^10^.

**Figure 1:**
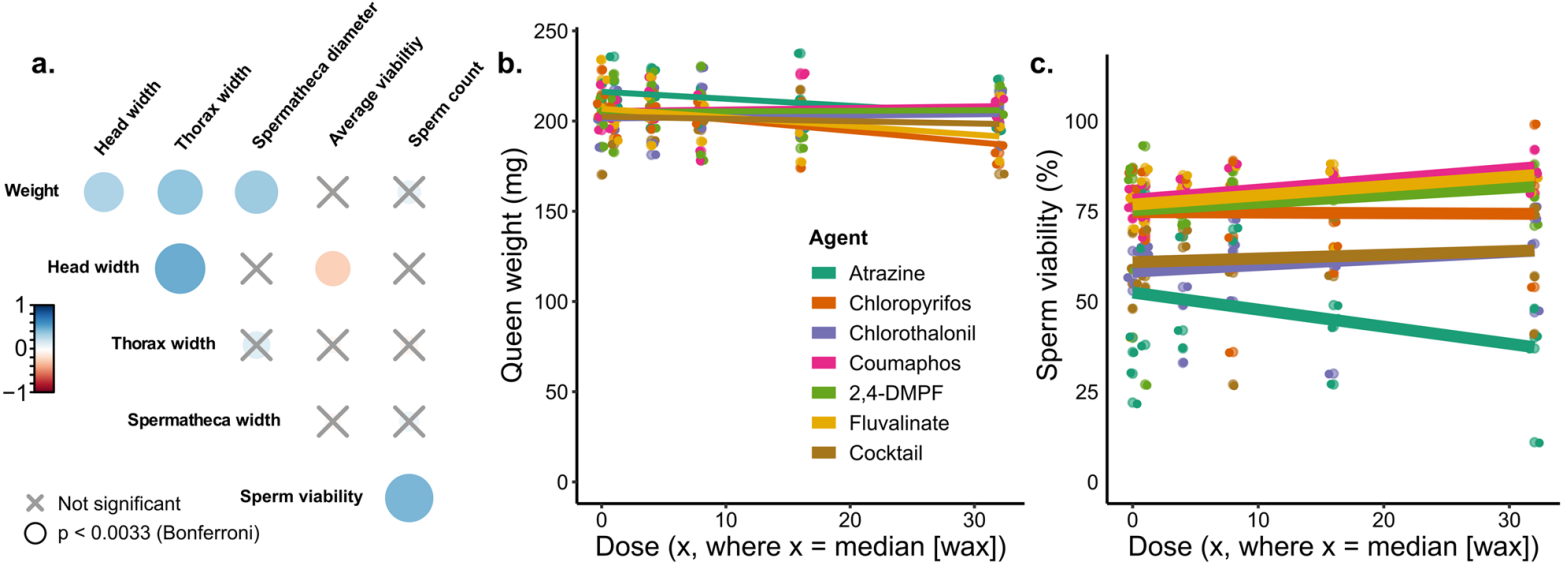
*Evaluating the effect of pesticide doses on queen quality metrics*. N = 4 queens were exposed topically (2 µl to the thorax, acetone solvent) to each pesticide and each dose, for a total of 168 queens. a) Queen quality metrics were recorded after exposure. The colour bar is proportional to the Pearson correlation coefficients. Neither queen weight (b) nor sperm viability (c) depends on dose or pesticide. See Table 2 for a complete statistical summary.

A total of 168 queens were analyzed, although four queens perished during the experiment for reasons not associated with a specific pesticide or dose (Supplementary Table S1). We first checked that the fertility and morphometric measurements of these queens was consistent with previous research (Fig. 1a). As expected, queen weight was significantly and positively correlated with head and thorax width, as well as spermathecal diameter (Spearman correlations, *p* < α = 0.003 with Bonferroni correction), as previously demonstrated^36,37^. Moreover, sperm viability and sperm count were also positively correlated (ρ = 0.507, *p* = 5.1 × 10^–12^). Next, we tested if pesticide treatment affected queen mass or sperm viability, as these are quality metrics which have been previously been shown to change in response to abiotic stressors^1,2,6^. We found no dose–response relationships of any of the compounds in relation to queen weight or sperm viability after correcting for multiple hypothesis testing (Fig. 1b and c; Table 2).

**Table 2:** Summary statistics for dose–response queen exposures*.

Agent	R^2^	F	df_1_	df_2_	P value
Chlorpyrifos	0.0232	1.18	3	20	0.342
2,4-DMPF	− 0.0335	0.751	3	20	0.534
Fluvalinate	− 0.0454	0.696	3	18	0.567
Coumaphos	0.294	4.05	3	19	0.022
Chlorthalonil	− 0.119	0.218	3	19	0.882
Atrazine	0.116	2.01	3	20	0.146
Mixture	− 0.142	0.0878	3	19	0.966

*Bonferroni correction: *p* < α = 0.007 for significance.

**Figure 2:**
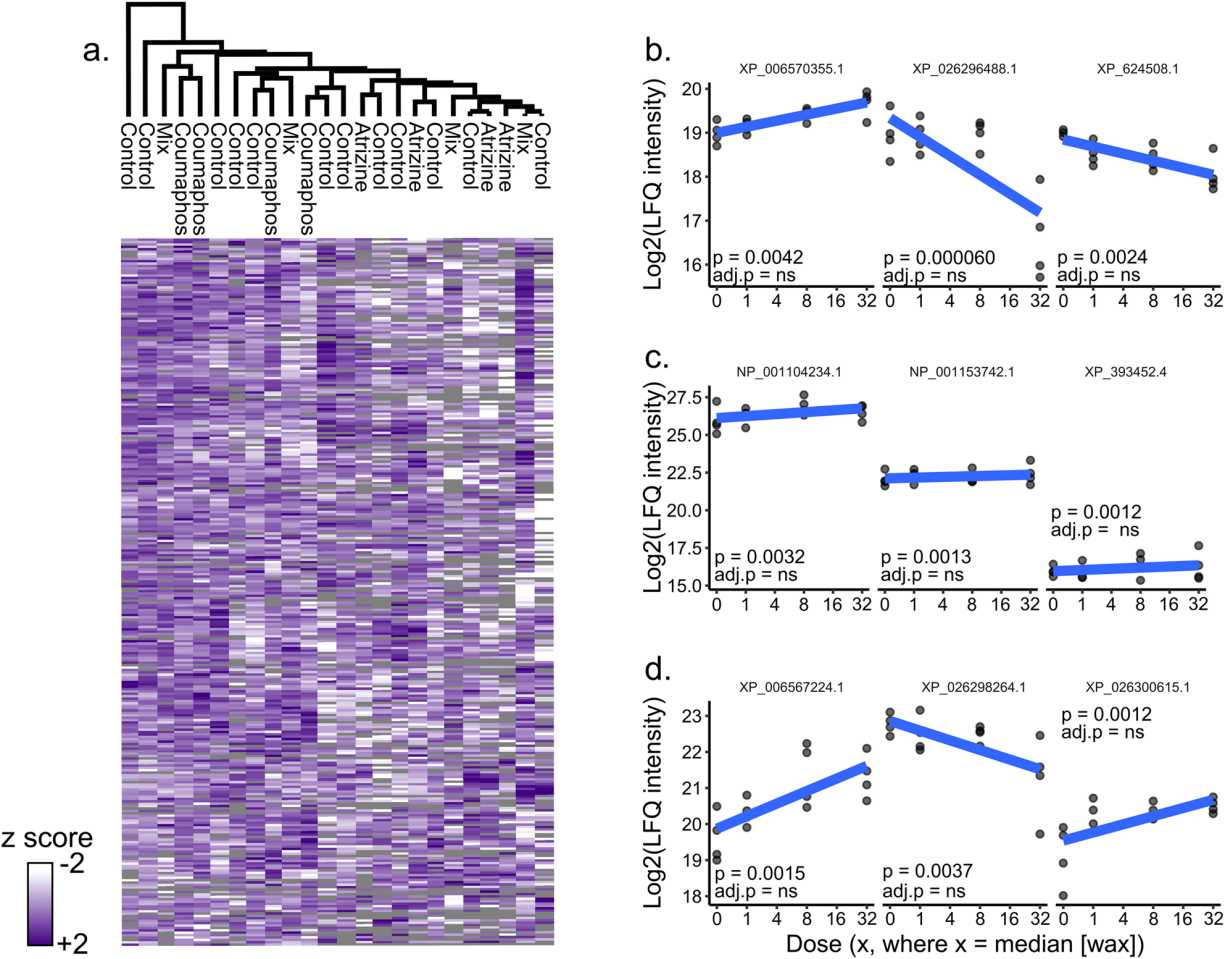
*Evaluating the effect of pesticide exposure on queen fat body protein expression*. We performed label-free quantitative (LFQ) proteomics on proteins extracted from fat bodies of atrazine-, coumaphos-, and cocktail-treated queens dosed at 0x, 1x, 8 × and 32x. a) We found no differences between the highest pesticide dose (32x)^10^ and their acetone controls (10% Benjamini–Hochberg correction). Samples and proteins were clustered via Euclidian distance, 300 clusters, 10 iterations. Grey tiles indicate samples in which a protein was not identified. Top proteins linked to pesticide dose for atrazine (b), coumaphos (c), and the cocktail (d). None were significant after correcting for multiple hypothesis testing. Accessions indicate refseq IDs. ns = protein does not survive 10% FDR.

We hypothesized that queens would initiate a broad metabolic detoxification response following topical pesticide exposure, perhaps explaining a lack of phenotypic effect. To test this hypothesis, we conducted mass spectrometry-based proteomics on the fat body tissue, a major source of detoxification enzyme production^38^, of queens exposed to atrazine, coumaphos, and the cocktail treatment. We chose these specific treatments for analysis because, in addition to being a controversial endocrine disruptor^39,40^, atrazine is the major herbicide residue found in honey bee hive matrices^41^ and may affect invertebrate reproductive characteristics^42^. Additionally, coumaphos was the compound most strongly correlating (although still not statistically significant) with sperm viability, and the cocktail treatment offers the most realistic exposure scenario. We quantified 1568 protein groups in the fat body (1% protein and peptide FDR; Supplementary Tables S2 and S3), and for each treatment, compared protein expression in the highest dose (n = 4 at 32×) to the control (n = 4 at 0×). We observed no significant correlations between protein expression and pesticide identity or dose (analysis conducted using the limma package in R, see Supplementary File S1 for example R code) even at a relatively loose false discovery rate (10%, Benjamini–Hochberg correction; Fig. 2a–d).

**Figure 3:**
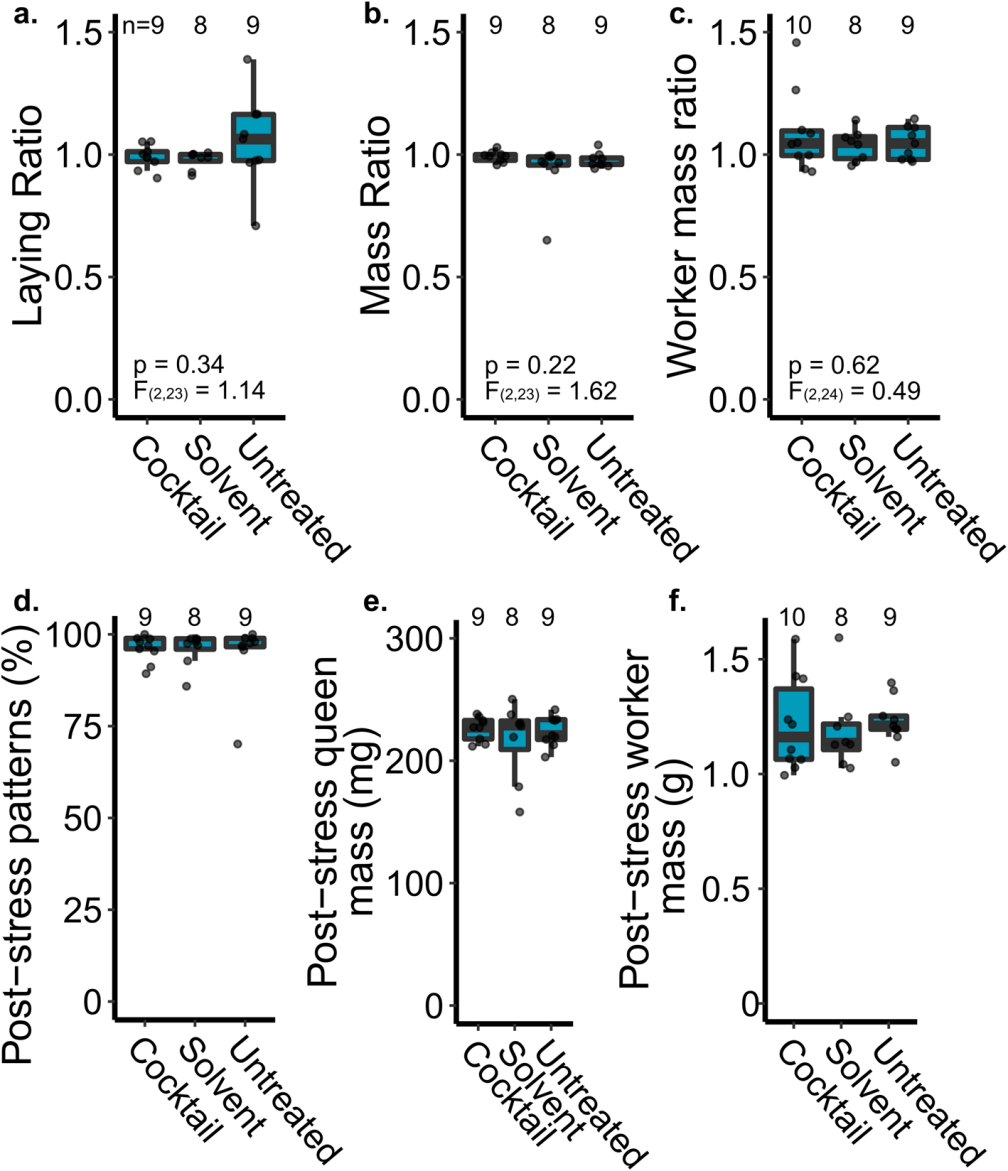
A field experiment evaluating the effect of topical pesticide cocktail treatment on queen performance. (a-c) We measured the queen’s laying pattern and mass immediately before and 2 weeks after topical pesticide exposure (2 µl, dosed at ~ 3,500 HQ, or 2.3 × where x = the median wax concentration). Ratios indicate the post-stress metric relative to the pre-stress measurement to account for variation between individual queens. Sample sizes and statistical parameters are indicated on the graphs. (d-f) Non-normalized post-stress metrics. Boxes represent the interquartile range, bars indicate the median, and whiskers span 1.5 times the interquartile range.

Queen mass and sperm viability are important fertility metrics, but impacts of pesticides on queen fertility could have a delayed response and require longer to manifest^11^. Moreover, some aspects of reproduction (e.g., laying pattern or vertical effects on progeny) are not captured by a laboratory dose–response experiment; therefore, we conducted a field experiment to measure additional queen and colony phenotypes before and after exposure to a pesticide cocktail. Since we aimed to test only the cocktail and were thus not limited by additional queens needed to test each component individually, we increased the complexity of the cocktail by the addition of fenpropathrin (insecticide), pendimethalin (herbicide), and azoxystrobin (fungicide)—three additional wax pesticide residues which were previously detected in > 20% of beeswax samples^10^. We found no effect of pesticide treatment on the queen’s egg laying pattern, queen mass, or mass of callow workers (linear model; sample sizes, F statistics, degrees of freedom, and p values are shown on the figures; Fig. 3a–c). We analyzed these data as ratios (post-treatment:pre-treatment) to account for individual variation between the queens’ baseline characteristics, but we also confirmed that the absolute parameters were in the expected range (Fig. 3d–f). The majority of queens had laying patterns near 100% coverage within the measured patch (80% or lower is considered to be a ‘poor brood pattern^43^’, which applied to only one of our queens), all but two queens had masses > 200 mg (within the range of previously published data^44,45^), and average callow worker mass was 107 mg (again, similar to previously reported data^46^; Fig. 3d–f).

**Figure 4:**
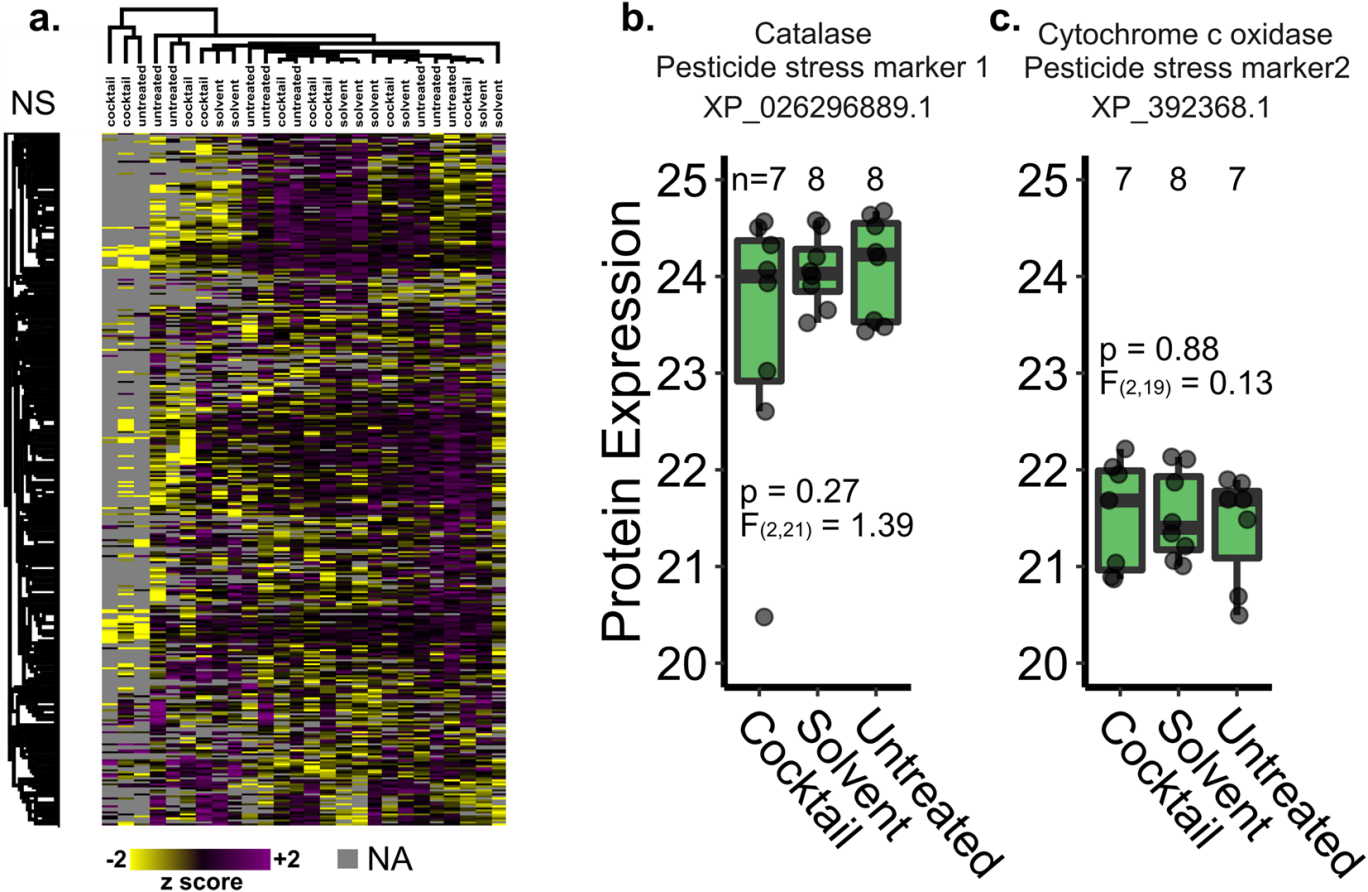
Protein expression in spermathecal fluid of queens treated with a pesticide cocktail. Queens were reintroduced to colonies after topical treatment (2 µl, dosed at 3,500 HQ) and sacrificed for analysis after 2 weeks. No proteins were differentially expressed at a global scale (limma, 10% FDR) (a) and the previously proposed pesticide stress biomarkers were also not differentially expressed (linear model; b & c). Samples and proteins clustered via Euclidian distance, 300 clusters, 10 iterations. Boxes represent the interquartile range, bars indicate the median, and whiskers span 1.5 times the interquartile range.

We previously proposed candidate pesticide stress biomarkers expressed in the spermathecal fluid after topical pesticide exposure^47^: Catalase (XP_026296889.1) and Cytochrome c oxidase (XP_392368.1). We ultimately aimed to use these candidate markers to detect pesticide stress, among other stressors, in failed queens. Here, we evaluated proteins expressed in the spermathecal fluid in order to determine if these candidate biomarkers remain present in the expected expression pattern (elevated in pesticide-stressed queens) despite the longer post-stress recovery period relative to our previous experiment in which they were identified (2 weeks vs. 2 days)^47^. We identified 3,127 protein groups (1% protein and peptide FDR; Supplementary Table S6) but found no differentially expressed proteins in the spermathecal fluid of queens treated with the pesticide cocktail compared to the untreated or solvent-treated controls (Fig. 4a; 10% Benjamini–Hochberg FDR). We also examined expression patterns of the candidate stress biomarkers specifically, but these too were not differentially expressed among groups (linear model, Fig. 4b and c), precluding their realistic utility as a diagnostic tool.

## Discussion

We report that adult queen health and sperm viability are likely unaffected by most pesticide residues commonly encountered through contact with beeswax. We report no substance-related effects from any of the six tested pesticides or their mixture on queen morphometrics, stored sperm quantity, or sperm viability at any dose levels (Fig. 1). The herbicide atrazine caused the largest reduction in overall sperm viability across tested compounds, but even this effect was not statistically significant (Table 2). Interestingly, the treatment mixture of all pesticides combined at their highest dose-level resulted in lower queen sperm viability (mean: 62.0% SD: ± 16.3%), which was lower relative to all other individual pesticides except for the herbicide atrazine (mean: 34.1% SD: ± 15.9%), although this relationship was also not statistically significant (Table 2). We observed no interactions (*i.e.*, additivity, synergy, or potentiation) among pesticides in our cocktail treatment. These data suggest that acute exposure to pesticide concentrations commonly found in wax are not likely to adversely impact queens.

The highest exposure examined in this study was 32 times the respective median detection within beeswax from commercial colonies for each respective chemical (Table 1)^10^. These test concentrations exceeded maximum detections for all chemicals except chlorothalonil and 2,4,-DMPF, and the combination of all chemicals at these concentrations did not impart a measurable effect on tested queens. The exposures used in this experiment are also likely to be far above the theoretical pesticide exposures faced by an individual queen from contact from contaminated beeswax inside of a colony, because it can be assumed that only a portion of the residues present within beeswax are freely transferable to a queen’s cuticle. However, the dynamics of in-hive pesticide movement and how they shape colony exposure remain largely understudied, including how sub-lethal chronic exposure may affect a queen over the course of her lifetime.

Our laboratory results identifying no effect of topical exposure on queen quality metrics were corroborated by our field observations, where we again did not identify any impact of pesticide cocktail exposure on queen performance or average mass of her adult progeny (Fig. 3). Furthermore, we did not observe exposure-associated ‘queen events’ in the weeks following reintroduction to their hives. Although it is possible that our observation period (2 weeks post-stress) was not long enough to observe changes in performance, we think this is unlikely because topical exposure of a neonicotinoid pesticide to queens has been previously shown to reduce sperm viability within 1 week^1^, and other abiotic stressors can affect queen quality within days^2,6,48^. Together, these data suggest that topical pesticide exposure, such as what may occur from contact with wax, is not likely to be an important exposure route for queens.

The honey bee queen invests considerable metabolic resources in maintaining viable sperm within the spermatheca, and stored sperm is an important indicator of honey bee queen longevity^4–6,49^. Our findings indicate that adult queens may have a robust capacity for protecting sperm from the potentially deleterious impacts of contact exposure to certain pesticides commonly found within commercial colonies. This is surprising, since queens should be under relatively low selective pressure for detoxification abilities, as opposed to workers, which directly interact with and consume a wider range of phytochemicals and indeed, pesticides. Queens are, however, under strong selection for processes that promote lifespan and mitigate damage to stored sperm—for example, by limiting the presence of reactive oxygen species (ROS)^5^. ROS are by-products of small molecule detoxification^50^, and efficient ROS detoxification may further limit adverse effects of exposure. Additionally, many other detoxification enzymes (*e.g.* some esterases and hydrolases) are multifunctional with other roles in metabolism; indeed, some research in *Drosophila* suggests that constitutive expression of metabolic genes other than classical detoxification enzymes (e.g. cytochrome p 450 or glutathione-S-transferase) are an important part of the detoxification response^51^. Therefore, queens could have gained pesticide tolerance through indirect selection on other processes.

One study, using similar topical queen exposures, also found no effects on sperm viability at exposures up to 100× the median detection for the miticide coumaphos^1^. Sperm viability of drones, too, is unaffected by topical exposure to a range of miticide doses^52^. Miticides and their derivatives were the most abundant compounds found in beeswax^10^. Therefore, these findings together with our negative results highlight that queen losses previously associated with residues in beeswax^10^ are likely not the result of direct toxic effects on queen or sperm health and instead may arise from other indirect social effects. One explanation may be that workers inhabiting hives that contain highly contaminated beeswax or pollen may perceive their environment in a way that makes them more likely to initiate the processes which result in queen events (queen replacement and death). Contrary to many other animals that exhibit increased cooperation under stressful conditions, it has been previously shown that workers that were starved during larval development emerge as adults with a reduced response to queen mandibular pheromone^53^. Queen pheromones are an important signal for modulating cooperative behaviors in workers such as foraging and brood rearing^3^, and stressor-mediated changes to pheromone signaling deserve future attention. Oral pesticide exposure has been shown to influence queen nutrition during development^21^, and it is unknown how pesticide exposure may interact with the queen pheromone production and their perception by workers. Furthermore, the most visible phenotypes under investigation as indicators of queen health (e.g., brood pattern and colony population) are not solely under queen control, and brood viability can be impacted by other stressors not relating to queen quality^54^. Finally, in this study, we did not investigate potential indirect effects of queen fertility via mating with pesticide-exposed drones. Some pesticides can reduce drone fertility and survival^31,32^; however, the impact of field-realistic pesticide cocktails occurring in hive products on drone fertility has not yet been investigated. While drone sperm viability declines with exposure to thiamethoxam and clothianidin^31^, it is not clear if and how this may affect queen fertility, since the queen acquires a ~ 20-fold excess of sperm during mating and only viable sperm can migrate into the spermatheca for storage^3^. However, it is possible that the longevity of viable sperm could be affected by drone exposure, and this is an important area of future research.

We previously observed that at a lower dose of the same pesticide cocktail (HQ 512, lower than any tested dose here), many proteins were differentially expressed in the queen’s spermathecal fluid within 2 days after exposure^47^. Furthermore, research by Chaimanee et al*.* identified acute detoxification responses as little as 1 day after exposure to imidacloprid, coumaphos, and amitraz^1,34^. Here, however, we found that 2 weeks after exposure to a much higher dose (HQ 3500) there were no discernable expression differences (Fig. 4). This suggests that, at least for the compounds we tested here, queens may have a rapid detoxification response and quickly clear the compounds before harmful effects are realized. Alternatively, this could be an example of hormesis, whereby toxic substances can exhibit biphasic biological responses, with seemingly disproportionate impacts of low doses^55^. Under hormesis, low doses of substances that are toxic at higher doses can stimulate beneficial or adaptive responses, which could explain the stronger biological response observed at the low dose we previously tested. Unfortunately, regardless of the underlying drivers, we found that the protein biomarkers for pesticide exposure that we previously proposed are not sufficient to indicate exposure in a realistic scenario. Given that we found no direct effect of topical exposures on queen quality, a different strategy will be needed to 1) understand the basis for the relationship between high residue concentrations and queen events, 2) develop an exposure method that reflects that relationship, and 3) analyze queens exposed using those methods to suggest new candidate biomarkers.

## Conclusion

Our laboratory exposure data and field observations suggest that contact exposure to pesticide concentrations commonly found in wax are unlikely to directly impair queen quality. While we acknowledge that results of chronic exposure over the course of a queen’s lifetime may differ, we tested a range of doses far exceeding what a queen should ever encounter, and still we found no effect on queen quality metrics and fat body protein expression. Combined with observing no change in queen performance within colonies after exposure to the complete pesticide cocktail, these findings suggest that previous associations between residue concentrations and ‘queen events’ are more likely to be driven by indirect effects on the queen through exposed workers.

## Methods

### Queen dose–response exposures and dissections

Queens with unknown relatedness were purchased from Wilbanks apiaries and banked for approximately 2 weeks prior to the start of the experiment. Queens were placed in lots of 24 queens, of which four queens were exposed to one of six ascending treatment doses (0×, 1×, 4×, 8×, 16×, or 32×) of six different compounds diluted in acetone, where x = the median concentration in wax as reported by Traynor et al*.*^10^ We used worker LD_50_ values previously reported in Traynor et al.^10^ when calculating HQ values for each test mixture. Worker toxicity values were used because queen LD_50_ values have not been established for most pesticides. The LD_50_ values provided in Traynor et al.^10^ include composite endpoints as an average across multiple worker acute toxicity values and some of the LD_50_ values include are non-definitive values, where no effects were not observed across all test concentrations. The LD_50_ values from Traynor et al.^10^ were used In order to simplify cross comparison between treatment HQs and the HQ values found in wax from commercial colonies. A further 24 queens were exposed to a cocktail blend of all six compounds. The cocktail was mixed such that each compound was present in the same relative concentration as reported by Traynor et al*.*^10^ and at the same dosing scale as the individual compounds. See Table 1 for all doses. The six compounds we tested were the same as those used in the topical exposures for the field trial, except that we excluded fenpropathrin, azoxystrobin, and pendimethalin to simplify our experimental design.

Two microliters of pesticide solution diluted in acetone were applied to the thorax of each queen, which were observed for approximately 5 min and then stored in Benton cages with attendants, fondant, and water delivered by a damp dental wick in an incubation chamber kept at 34.5 ˚C. Queens were monitored and water replenished periodically during this time, approximately every 24 h. 48 h following exposure, queens were anesthetized with CO_2_ from sublimated dry ice until immobile. Each queen was removed from its cage, weighed, pinned to a dissection stage ventral-side up, and photographed. Then the abdominal tegument was separated at the 6th abdominal tergite and the spermatheca was gently removed, which was itself photographed prior to immersion in 1 mL of Buffer D (17 mM D-glucose, 54 mM KCl, 25 mM NaHCO_3_, 83 mM Na_3_C_6_H_5_O_7_). The queen was then stored in a 1.7 mL centrifuge tube at  − 80 °C for future fat body proteomics analyses.

### Sperm viability analysis

The spermatheca was ruptured with a pair of ridged forceps and the released contents mixed, transferred into a 1.7 ml centrifuge vial, and vortexed gently for 15 s. An aliquot of 200 µl was transferred to a 1.0 ml amber chromatography vial containing 2.0 µl propidium iodide solution and Sybr 14 dye from a Thermo-Fisher Live-Dead Sperm viability kit essentially as previously described^56^. This aliquot was vortexed to mix and capped, allowing it to incubate at room temperature until all queens were processed, which took approximately 2.5 h.

After the spermathecal contents had incubated with the dyes for at least 10 min and no more than 3 h, 20 µl was transferred to a Nexcelom Cellometer counting chamber for spermatozoa count and viability imaging essentially as previously described^11,56^. The average of the three reads was taken for the measures of total spermatozoa concentration, and the viability was recorded as the ratio of live to total spermatozoa.

### Morphometric analysis

Photographs of queen morphometric measurements were analyzed using ImageJ. The width of the head was taken at the widest point perpendicular to the body axis in a line passing over the frons. The width of the thorax was taken as the width of the mesothoracic sternite directly parallel to a line intersecting the tegulae. Measurements were converted from pixels to mm by comparison to a 0.1 mm microscopic rule.

### Pesticide stress field trial

Honey bee colonies were established from imported Tasmanian packages as part of a previously described experiment^57^. Briefly, packages were installed in standard 10 frame deep hive bodies, supplied with pollen and syrup, and after 1.5 months (two brood cycles), we split the colonies into 30 three-frame nucleus colonies (nucs), each with one frame of honey, one frame with open brood, and one frame with capped brood. We supplied each nuc with a frame feeder for light syrup (~ 35% sucrose) and a ½ lb pollen patty (15% protein), which we fed continuously throughout the duration of the field trial. All nucs were kept in a single apiary in Richmond, Canada.

Each nuc was supplied with a queen imported from California (all from a single shipment with unknown relatedness, supplied by Olivarez Honey Bees Inc.). Caged queens were placed between two frames with the cage screen facing down and allowed to acclimate for 3 days, at which time the queens were released. 2 days later, we checked the nucs for queen acceptance and those that were rejected were supplied a new queen from the same batch.

Once each queen had been laying eggs for at least 2 weeks, we evaluated their laying patterns, as previously described^57^, by locating a patch of approximately 100 eggs and recording how many cells within that patch were apparently missed (i.e., the cell was not otherwise occupied but lacked an egg). We avoided patches at the edge of the brood area. If a patch included an occasional cell with a newly eclosed larva, it was counted as ‘laid’ since it was likely that the eggs were on the verge of hatching. This method does not distinguish between eggs that were laid and then cannibalized by workers; however, since all colonies were fed supplemental protein, egg cannibalization should be linked to developmental deficiencies or failure to hatch rather than nutritional stress, which is a desirable feature to which our method should be sensitive. We repeated this procedure 2 weeks after the queens were exposed to pesticide treatments in order to calculate a change in laying pattern (the ratio of the fraction of cells laid post-stress relative to pre-stress).

On the day that we evaluated laying pattern, we caged queens with five attendants and candy, then transported to the laboratory where a colleague not otherwise involved in the study briefly anesthetized the queens with carbon dioxide, weighed them on an analytical balance, and randomized them into three treatment groups (untreated, 2 µl acetone, and 2 µl cocktail dissolved in acetone), keeping the lead experimenter blind to their assignments. We administered the cocktail using the same method as described for the dose–response experiment but delivered the cocktail at a Hazard Quotient (HQ) of ~ 3,500, which is the hazard level that has been previously found to be associated with ‘queen events’ (queen loss or supersedure) (Table 3)^10^. This corresponds to 2.3 × the median wax HQ found in Traynor et al.^10^. During the experiment, four of the thirty original queens perished (unrelated to treatment group; one cocktail, two solvent, and one untreated queen). We measured the queens’ egg laying pattern and wet weight before and after pesticide treatment (Supplementary Table S4), as well as the average wet weight of newly emerged workers born from eggs laid before and after the queens were stressed (Supplementary Table S5). This pesticide cocktail is the same as used in the dose–response experiment, but with the addition of fenpropathrin, pendimethalin, and azoxystrobin (three additional compounds found in wax), mixed and adjusted by serial dilution in the same relative proportions as reported by Traynor et al*.*^10^. This is the same mixture as was reported in McAfee et al*.*^47^, but applied at a higher dose (Table 3). Treatments were administered topically to the queen’s thorax, then queens were returned to their cages with the workers, transported back to the apiary, and re-introduced to their respective colonies, but remained caged for 2 days before release. 2 weeks post-stress, laying patterns were again evaluated, the queens were transported to the laboratory, anesthetized, weighed, and sacrificed for dissection. Spermathecae were removed from the abdomen with forceps and blotted dry on a Kimwipe, then clean forceps were used to gently remove the tracheal net surrounding the spermatheca. The spermatheca was then lysed in an Ependorf tube containing 100 µl Buffer D and spermathecal fluid proteins were extracted for mass spectrometry analysis exactly as previously described^58^.

**Table 3:** Composition of the 9-component cocktail used for the field trial, derived from Traynor et al*.*^10^.

Pesticide	LD_50_	ppb	HQ
Fluvalinate	4.32	9840.2	2277.8
Coumaphos	5.93	2153.0	363.1
2,4-DMPF	75	694.1	9.3
Chlorothalonil	111	824.2	7.4
Chlorpyrifos	0.0762	6.2	80.9
Atrazine	98.5	12.3	0.1
Fenpropathrin	0.05	38.4	767.1
Pendimethalin	74.9	12.1	0.2
Azoxystrobin	112	11.6	0.1
Cocktail			3506.0

We collected newly emerged (callow) workers from each colony 4 weeks after the beginning of the experiment and 4 weeks after the queens were stressed. It takes an average of 21 days for worker eggs to develop into adults^3^; therefore, callow workers collected 4 weeks after the beginning of the experiment developed from eggs laid approximately 1 week after the experiment began (i.e., before the queens were stressed). Likewise, callow workers collected 4 weeks after the queens were stressed developed from eggs laid one week post-stress. The 2-day caging period immediately following the queen pesticide exposures provided time for the stress response to manifest and provided a short brood break to guard against overlap between pre- and post-stress newly emerged bees due to potential variation in developmental times.

We collected 9–12 callow workers per colony per time point in order to calculate a change in average mass at emergence. Callow workers are easily recognizable due to their light grey color, soft bodies, and inability to fly. We recorded the number of workers and wet weights on an analytical balance.

### Proteomics analysis

Proteins were extracted, digested, and purified from spermathecal fluid exactly as previously described^57,58^. Briefly, sperm cells were spun down from the Buffer D-diluted spermathecal fluid solution and soluble proteins in the supernatant were precipitated with acetone. The pellets were washed and resuspended in urea digestion buffer (6 M urea, 2 M thiourea, in 100 mM Tris, pH 8). The proteins were reduced, alkylated, then digested with 0.2 µg of Lys-C (3 h, room temperature) followed by 0.2 µg of trypsin (overnight, room temperature, solution diluted with 4 volumes of 50 mM ammonium bicarbonate). Peptides were desalted using in-house made C18 STAGE-tips, dried, suspended in Buffer A (0.1% formic acid, 2% acetonitrile), and quantified using a Nanodrop (280 nm absorbance). One µg of peptides were injected on a Thermo easy-nLC 1000 liquid chromatography system coupled to a Bruker Impact II mass spectrometer. Sample orders were randomized prior to loading, and instrument parameters were set exactly as previously described^47^. We followed the same procedure for analysis of the queen fat bodies, except that protein was extracted into 6 M guanidinium chloride (in 100 mM Tris, pH 8) using a Precellys homogenizer with ceramic beads. We digested approximately 25 µg of protein per sample using 0.5 µg of Lys-C and trypsin.

Raw mass spectrometry data were searched using MaxQuant (v 1.6.1.0) exactly as previously described^47^. We used the most recent honey bee canonical protein database available on NCBI (HAv3.1, downloaded November 18th, 2019) with honey bee pathogen sequences added. Protein and peptide identifications were filtered to 1% FDR based on the reverse hits approach. All specific search parameters are available within the mqpar.xml file included in our data repository (see Data Availability).

### Statistical analysis

We analyzed laying pattern ratio, queen mass ratio, and worker mass ratio data using linear models in R (v3.5.1) with queen treatment included as a fixed effect. Queen sperm viability and morphometric analyses were conducted using R (v3.6.0). Because of the disparity between the number of tests to be run and the relatively small number of queens per group (n = 4), we used principal components to reduce the number of variables under consideration. Namely, we generated the first principal component of all the correlated morphometric variables and used this as a final measurement of queen size. Queen size was used as a covariate in the final analyses. Additionally, we would not expect that sperm count would change as a result of this treatment, as it was applied after mating and non-viable sperm do not appear to be destroyed in the spermatheca^59^. Thus, we also used total sperm as a covariate in a final model testing the effect of topical agrichemical treatment on sperm viability.

For spermathecal fluid proteomics data, protein intensities (‘LFQ intensity’ columns from the MaxQuant output) were first log2 transformed, then reverse hits, contaminants, protein groups only identified by site, and protein groups without at least three defined values per treatment group were removed. Differential expression analysis was performed using limma() (example code is provided, see Data Availability) and a Benjamini–Hochberg multiple hypothesis testing correction to 10% FDR. We analyzed the queen fat body proteomics data in the same way, except queen fat body proteins were filtered to include only those that were identified in at least 3 of the 4 biological replicates at each dose. We analyzed expression of individual candidate biomarkers (six proteins) in the spermathecal fluid data using a linear model. Heatmaps were generated using Perseus v1.6.1.1 (clustered via Euclidian distance, 300 clusters, 10 iterations).

## Supplementary Information

Supplementary Information 1.

Supplementary Information 2.

Supplementary Information 3.

Supplementary Information 4.

## Data Availability

All raw proteomics data and search results have been deposited to the MassIVE proteomics repository (massive.ucsd.edu; field experiment data: accession MSV000086862; dose–response experiment data: accession MSV000087091). Phenotypic data for the queen dose–response topical exposure experiment are available in Supplementary Table S1. Queen fat body protein expression data and metadata are in Supplementary Tables S2 and S3, respectively. Data from the queen exposure field trial is available in Supplementary Tables S4 and worker mass data is in Supplementary Table S5. An example R code for the limma protein expression analysis of fat bodies is available as Supplementary File S1. R code for the dose–response exposure analysis and field trial data analysis are available as Supplementary Files S2 and S3, respectively.
